# The clinical utility of serum prealbumin levels as a prognostic marker in patients with hepatocellular carcinoma undergoing transcatheter arterial chemoembolization: a meta-analysis of 2,996 patients

**DOI:** 10.3389/fonc.2026.1782104

**Published:** 2026-03-12

**Authors:** Weiming Yu, Junjie Zhang, Qunfeng Xia

**Affiliations:** Department of Hepatobiliary and Pancreatic Surgery, Fuyang Campus of Zhejiang Provincial People’s Hospital (The First People’s Hospital of Fuyang), Hangzhou, China

**Keywords:** hepatocellular carcinoma, overall survival, prognosis, serum prealbumin, transarterial chemoembolization

## Abstract

**Introduction:**

Hepatocellular carcinoma (HCC) is a leading cause of cancer death globally. Transarterial chemoembolization (TACE) is key for unresectable HCC, but patient outcomes vary, necessitating reliable prognostic markers. Although preoperative serum prealbumin (PAB) reflects nutrition and liver function and shows prognostic potential in single-center studies, high-level evidence is lacking. This meta-analysis aimed to systematically confirm the prognostic value of baseline serum PAB in HCC patients undergoing TACE.

**Materials and methods:**

Relevant publications up to February 3, 2026, were retrieved from PubMed, The Cochrane Library, Embase, Web of Science, CNKI, Wanfang, VIP, and CBM databases. Fixed-effect or random-effects models were used to calculate the pooled hazard ratio (HR) for overall survival (OS) and odds ratio (OR) for clinicopathological characteristics associated with serum PAB. Sensitivity and subgroup analyses investigated heterogeneity and assessed result stability. Publication bias analysis was conducted to evaluate the reliability of the meta-analysis findings.

**Results:**

Eight datasets from five studies were analyzed. Lower baseline PAB was significantly associated with poorer OS (pooled HR for high vs. low PAB = 0.64, 95% CI: 0.59–0.70, P<0.0001; random-effects model, I^2^ = 0%). Higher PAB correlated with favorable features: earlier BCLC stage (OR = 2.23), better Child-Pugh class (OR = 5.36), absence of vascular invasion (OR = 0.44) or extrahepatic metastasis (OR = 0.22), smaller tumor size (OR = 0.51), single tumor (OR = 1.39), and lower AFP (OR = 0.56) (all P<0.05 except single tumor P = 0.020 and AFP P = 0.006). No significant association was found between prealbumin level and hepatitis B virus (HBV) infection status (P = 0.630). Subgroup and sensitivity analyses supported these findings, with no significant publication bias.

**Conclusion:**

The analysis of 2,996 patients confirms that lower baseline serum PAB is significantly associated with poorer OS and unfavorable clinicopathological features in HCC patients receiving TACE. Further prospective and multi-center studies are needed to validate its clinical utility.

## 1 Introduction

Hepatocellular carcinoma (HCC) is the most common primary liver malignancy, posing a significant public health challenge worldwide. According to the latest global cancer statistics (GLOBOCAN 2022), the annual new cases of HCC exceeded 900,000, and the annual death cases were approximately 830,000. Its incidence and mortality rates rank sixth and third, respectively, among all cancers (1). Despite progress in screening, diagnosis, and local treatment, most patients are diagnosed at the middle or advanced stage and lose the opportunity for radical surgical resection or liver transplantation (2).

For patients with inoperable advanced HCC, the treatment goal aimed at cure is limited, so the treatment goal shifts to prolonging survival and improving quality of life. In this context, local interventional therapy such as transarterial chemoembolization (TACE) has become the standard treatment option for patients with Barcelona Clinic Liver Cancer (BCLC) stage B (intermediate) liver cancer and has been applied in some selected advanced patients (2, 3). However, clinical practice has shown significant heterogeneity in the treatment response and long-term survival outcomes of patients to TACE (4). This difference is not only due to the characteristics of the tumor itself (such as size, number, and presence of vascular invasion), but also to host factors, especially the liver reserve function and systemic inflammatory-nutritional status of the patients (5). Therefore, finding individualized prognostic indicators that are simple, accurate, and can be evaluated before treatment is of core clinical value for optimizing treatment decisions (such as patient stratification, combined treatment selection) and achieving precision medicine.

Serum prealbumin, a protein synthesized by the liver, has a short half-life and sensitively reflects the hepatic synthetic function and the nutritional status of the body (6). Its prognostic value has been confirmed in various malignant tumors (7–9). A recent meta-analysis integrating evidence from 11 studies involving 7,442 patients clearly showed that a prealbumin level lower than normal before surgery was independently associated with a significantly shorter overall survival and recurrence-free survival (RFS) in HCC patients undergoing radical liver resection (10). This finding emphasizes the importance of nutrition and inflammatory status in the prognosis assessment of HCC.

In recent years, relevant studies have begun to specifically focus on the predictive role of prealbumin levels before treatment on the survival outcomes of HCC patients receiving TACE therapy (11–15). However, the prognostic significance of prealbumin, a biomarker reflecting both nutritional status and systemic inflammation, in HCC patients treated with TACE warrants comprehensive evaluation. In our systematic literature search, we found no meta-analysis that specifically has addressed the prognostic value of baseline serum prealbumin (PAB) in HCC patients receiving TACE. Therefore, this meta-analysis of 2,996 patients aims to consolidate and strengthen the existing evidence by systematically confirming, for the first time, the prognostic value of baseline serum PAB for overall survival (OS) and its correlation with key clinicopathological features in this specific patient cohort, thereby providing powerful support for its clinical application. It should be noted that although previous meta-analyses have confirmed the prognostic significance of preoperative PAB for HCC patients undergoing radical liver resection, patients receiving TACE treatment typically have a later tumor stage and worse baseline liver function, and their tumor biological behavior and treatment response patterns differ fundamentally. Therefore, an assessment specifically targeting this particular patient population is necessary and has unique value.

## 2 Materials and methods

### 2.1 Literature search

Two independent researchers searched the databases of PubMed, The Cochrane Library, Embase, Web of Science, China National Knowledge Infrastructure (CNKI), Wanfang, VIP, and China Biomedical Literature Service System (CBM) to collect all studies related to the correlation between serum prealbumin and the prognosis assessment of patients undergoing transarterial chemoembolization (TACE) treatment. The search period covered the earliest available records in the databases up to February 3, 2026. The main search terms included: (“Liver Neoplasms”[MeSH Terms] OR “Carcinoma, Hepatocellular”[MeSH Terms] OR “Hepatocellular Carcinoma”[Title/Abstract] OR “HCC”[Title/Abstract] OR “Liver Cancer”[Title/Abstract] OR “Hepatoma”[Title/Abstract]) AND (“Chemoembolization, Therapeutic”[MeSH Terms] OR “Transarterial Chemoembolization”[Title/Abstract] OR “TACE”[Title/Abstract] OR “Chemoembolization”[Title/Abstract]) AND (“Prealbumin”[MeSH Terms] OR”Prealbumin”[Title/Abstract] OR “PAB”[Title/Abstract] OR “Transthyretin”[Title/Abstract] OR “Nutrition Assessment”[MeSH Terms] OR “Nutritional Status”[MeSH Terms] OR “Prognostic Nutrition”[Title/Abstract] OR “Nutritional Index”[Title/Abstract]). To ensure the transparency and reproducibility of the research, we developed a detailed internal plan in accordance with the PRISMA (2020 version) guidelines (16) prior to the study commencement and adhered to it strictly. This plan clearly defined the research questions, search strategy, inclusion/exclusion criteria, data extraction items, the quality evaluation tool (NOS), and statistical analysis plan. The literature screening, data extraction, and quality evaluation were independently conducted by two researchers, and any disputes were resolved by cross-checking and arbitration conducted by a third researcher. The comprehensive search strategy is provided in **Supplementary File 1**.

### 2.2 Study selection

The inclusion criteria for this meta-analysis are as follows: (1) Patients pathologically diagnosed with hepatocellular carcinoma (HCC); (2) Measurement of serum prealbumin levels in each patient before TACE; (3) Patients who received initial (or first session) TACE as the primary treatment for HCC; (4) Studies reporting the correlation between serum prealbumin and overall survival (OS). The exclusion criteria are as follows: (1) Conference abstracts, letters, dissertations, case reports, reviews, or non-clinical studies; (2) Patients diagnosed with non-hepatocellular carcinoma; (3) Studies on repeat TACE, combined therapies or non-initial treatments; (4) Studies with fewer than 10 participants; (5) Studies reporting risk estimates obtained solely through univariate analysis; (6) Studies with insufficient data on estimated hazard ratio (HR) and 95% confidence interval (CI).

Two reviewers (YWM and ZZJ) independently screened the titles and abstracts of all retrieved records, then evaluated the full texts to confirm whether they met the inclusion criteria. All evaluations were conducted independently by the two reviewers, and their results were mutually verified. In case of disagreement, it was resolved through discussion; if necessary, consultation with a third reviewer (XQF) was sought.

### 2.3 Data extraction

The data extraction was independently carried out by two reviewers (YWM and ZZJ). To ensure accuracy, all extracted data were cross-checked. Any discrepancies were resolved through discussion, and when necessary, a third reviewer (XQF) was consulted. The extracted variables were as follows: the first author, publication year, research region, sample size, age, gender, treatment method, cut-off value, follow-up time, clinicopathological features of HCC patients (HBV infection; serum AFP level; Child-Pugh liver function classification; extrahepatic metastasis; tumor stage; tumor size; tumor number; vascular invasion), and hazard ratio (HR) with its 95% confidence interval (CI).

### 2.4 Quality assessment

The quality of these studies was evaluated using the Newcastle-Ottawa Scale (NOS) (17). This assessment system covers three aspects: the selection of research subjects (0–4 points), the comparability of the research population (0–2 points), and the evaluation of results (0–3 points), totaling nine points, with the highest score being 9. An NOS score greater than or equal to 6 points indicates a high-quality study. The specific scores of each study were detailed in **Table 1**.

**Table 1:** The basic characteristics of the enrolled studies.

Author	Year	Study region	Sample size	Treatment	Stage of tumor(BCLC stage)	PAB cut-off(mg/L)	OS HR(95% CI)	HR extraction method	Follow-up periods(months)	NOS score
Hou et al. (12)	2022	China	768	cTACE	A-C	170	0.671(0.554-0.812)	Report^1^	44	8
Lei (pre-PSM) et al. (13)	2023	China	402	cTACE	B-C	159	0.56(0.44-0.71)	Extract from the survival curve	120	8
Lei (post-PSM) et al. (13)	2023	China	144	cTACE	B-C	159	0.656(0.448-0.961)	Report^2^	113	8
Xu et al. (14)	2023	China	461	cTACE	A-C	170	0.71(0.56-0.91)	Report^3^	168	7
Xu et al. (15)	2024	China	1041	cTACE	A-C	170	0.67(0.56-0.82)	Report^4^	168	8
Yu et al. (11)	2019	China	324	cTACE	A-C	Male: Low:<120 Medium: 120–190 High: >190 Female: Low:<100 Medium: 100–170 High: >170	High vs Medium: 0.66(0.46-0.95) High vs Low: 0.41(0.26-0.64) Medium vs Low: 0.64(0.43-0.95)	Survival curve	68	7

cTACE, conventional transarterial chemoembolization. ^1^ HR has been adjusted: HBV, tumor size, number of tumors, ECOG/PS, BCLC, CTP stage, ascites, tumor location, vascular invasion, AFP, AST, Alb. ^2^ HR has been adjusted: age, sex, BCLC stage, CTP stage, vascular invasion, extrahepatic metastasis, AST, TBIL, ALB, HB, PLT, and PT. ^3^ HR has been adjusted: AFP, ALB, ascites, AST, BCLC, HBV, vascular invasion, tumor location, number of tumors, PS, PT, tumor size. ^4^ HR has been adjusted: AFP, Alb, ascites, AST, BCLC, liver cirrhosis, HBV, vascular invasion, tumor location, number of tumors, PS, PT, tumor size. pre-PSM: before Propensity Score Matching analysis. post-PSM: after Propensity Score Matching analysis.

### 2.5 Data analysis

All statistical analyses were conducted using Review Manager version 5.3 (available at http://www.cochrane.org/) and STATA version 16.0. We evaluated the impact of baseline serum prealbumin levels on adverse outcomes by summarizing the multivariable-adjusted hazard ratios (HR) and their 95% confidence intervals (CI) comparing the lowest and highest serum prealbumin categories; alternatively, these data were estimated through survival curves. For studies providing multiple comparative data sets, we apply the generic inverse-variance method to combine risk ratios, avoiding counting patients multiple times within the same study. Cochran’s Q test and Higgins’ I^2^ statistic were used to assess the heterogeneity of the included studies. We used the fixed-effects model (Mantel-Haenszel method) and the random-effects model (DerSimonian-Laird method) to calculate the combined HR or odds ratio (OR) and their 95% confidence intervals. If the p value is less than 0.10 or I^2^ is greater than 50%, this indicates significant heterogeneity; therefore, the random-effects model is adopted. Otherwise, the fixed-effects model is used. Subgroup analyses were conducted based on sample size, HR extraction method (direct extraction or estimation from survival curves), and follow-up time to explore potential sources of heterogeneity and verify the robustness of the results. Cochran’s Q test was assessed differences among subgroups in combined effect sizes. Publication bias was evaluated using funnel plots and Egger’s test (18), with a p-value <0.05 denoting statistical significance. During forest plot analysis, multiple independent comparisons derived from a single original study (such as analyses based on the total cohort and matched sub-cohorts) are included as separate entries. We used a random effects model to pool the data, which accounts for additional variation due to intra-study correlations, and evaluated each entry’s independent impact on the pooled results via sensitivity analysis. For studies where HRs were estimated from survival curves, we followed the standard methods recommended by Tierney et al. (19). Specifically, the Kaplan-Meier curves from the publications were digitized using WebPlotDigitizer (Version 4.6) to extract survival probabilities and the number of patients at risk at various time points. These data were then used to reconstruct individual patient data and calculate the HR and its 95% confidence interval (CI) using the methods described by Parmar and Tierney et al. We acknowledge that this method, while established, introduces measurement error compared to directly reported HRs, primarily stemming from the graph digitization process and the simplifying assumptions (e.g., proportional hazards) made during reconstruction. To assess the impact of this key methodological variation on the pooled results, it was included as a pre-specified variable in the subgroup analysis. Furthermore, the GRADE method was used to assess the quality of evidence for the primary outcome (overall survival).

## 3 Results

### 3.1 Research characteristics

**Figure 1:**
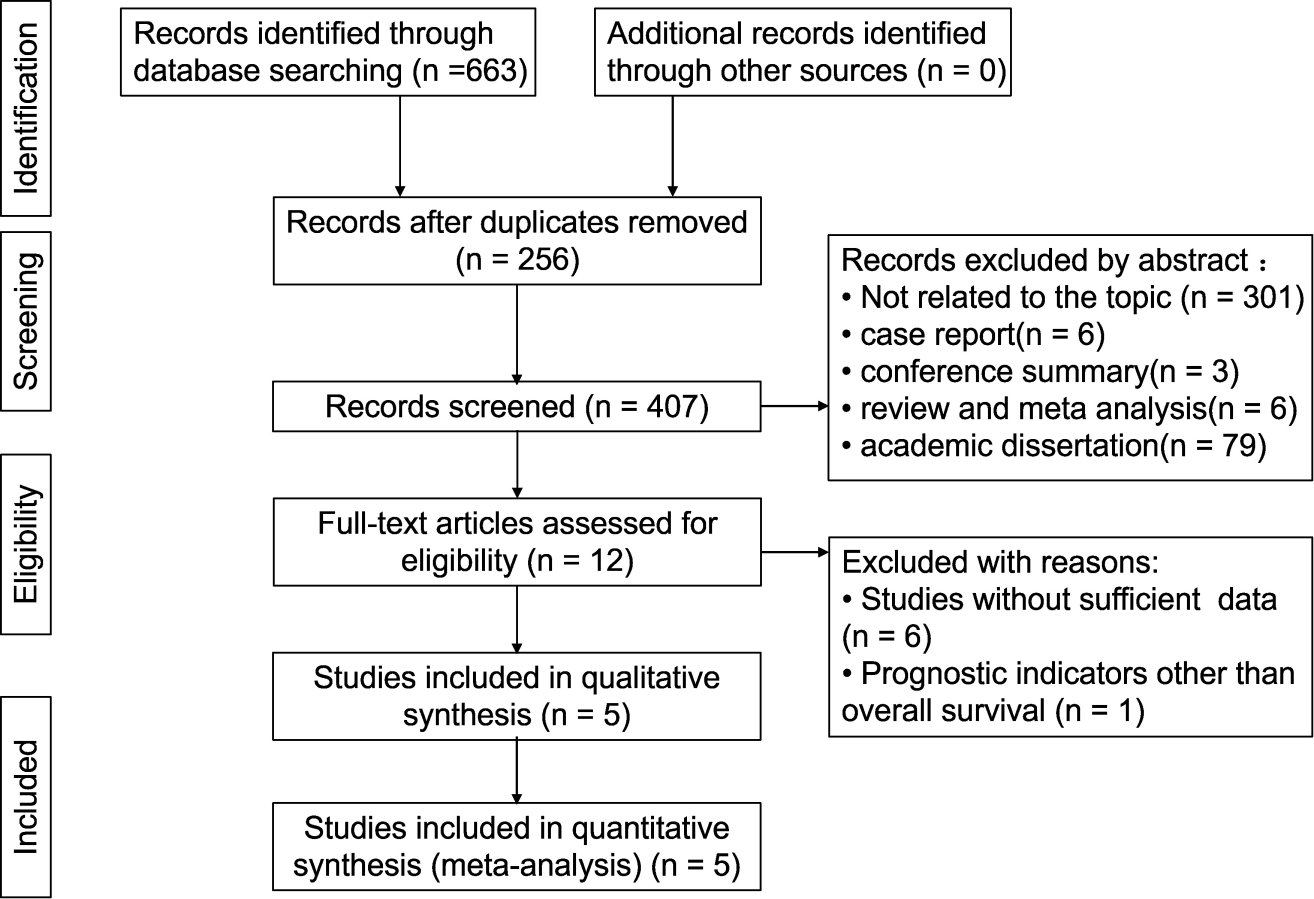
The flow diagram.

After a systematic search of 8 databases, a total of 663 articles were initially retrieved (PubMed: 43, Web of Science: 77, Embase: 144, Cochrane Library: 7, CNKI: 92, Wanfang Database: 163, VIP Database: 34, China Biomedical Literature Service System Database: 103), then 256 duplicate articles were excluded, leaving 407 articles for title and abstract screening. After excluding irrelevant topics, review and meta-analyses, letters, conference abstracts, case reports, academic dissertation, etc., 12 articles were evaluated through full-text reading. Among them, 7 studies were excluded due to either lack of survival data or inconsistent research objectives. Finally, this meta-analysis selected 5 articles that met the criteria. The flowchart of study selection is shown below (**Figure 1**).

Five studies published between 2019 and 2023 were included, all of which used a retrospective design, and all studies were conducted in China. It is notable that one of the studies included two independent cohort studies, and another study included a comparative analysis of 3 survival curves, resulting in a total of 8 comparison groups across these studies. Three studies directly extracted the multivariable-adjusted hazard ratio (HR) and its 95% confidence interval (CI), one study estimated the HR and 95% CI through survival curves and extracted data for two groups within the study, and one study obtained data for two groups within the study through both direct extraction and survival curve estimation. In all included studies, all patients received initial TACE treatment. In our meta-analysis, the 8 groups of data from the 5 studies demonstrated the association between serum prealbumin and OS, and one study demonstrated the association between serum prealbumin and PFS. The characteristics of the included studies are shown in **Table 1**.

### 3.2 Overall analysis results

#### 3.2.1 Serum prealbumin and OS in HCC

**Figure 2:**
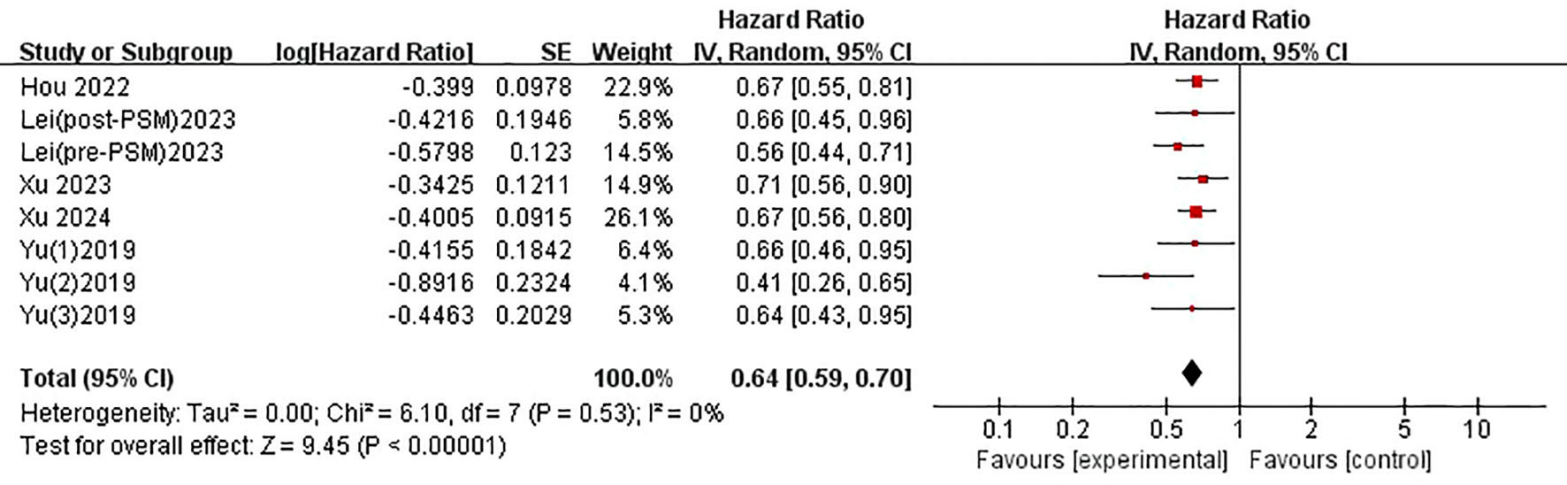
Association between PAB and overall survival of HCC.

The five included studies provided data for evaluating the association between serum prealbumin and overall survival (OS). Since two independent comparisons (based on the total cohort and matched sub-cohort analyses) from the same original study were treated as separate items, a random-effects model was used to pool the data. The results indicated no significant heterogeneity (P = 0.53, I^2^ = 0%). Our results clearly indicated that lower baseline serum prealbumin levels were significantly associated with poorer OS, with a combined hazard ratio (HR) of 0.64 (95% confidence interval [CI]: 0.59–0.70, P < 0.01) (**Figure 2**).

#### 3.2.2 Relationship between serum prealbumin and clinical pathological features of HCC

PAB and HBV infection: Two studies reported HBV infection (positive or negative), and the pooled results indicated that the baseline serum prealbumin level was not significantly associated with HBV infection in HCC patients (OR = 1.10, 95% CI = 0.74–1.63, P = 0.630) (**Table 2**).

**Table 2:** Association between high serum prealbumin levels and clinical pathological characteristics.

Parameter	N of studies	N of patients	Effect model	OR (95% CI)	P-value	Heterogeneity test	
						I^2^ (%)	P-value
HBV (+/−)	2	1092	Random	1.10(0.74-1.63)	0.630	56	0.080
BCLC stage(A-B/C)	3	1494	Random	2.23(1.60-3.11)	0.000	57	0.040
C-P classification(A/B)	2	1170	Random	5.36(3.29-8.72)	0.000	82	0.004
Vascular invasion(+/−)	2	1170	Fixed	0.44(0.34-0.58)	0.000	26	0.260
Extrahepatic metastasis(Yes/No)	3	1170	Fixed	0.22(0.14-0.35)	0.000	0	0.560
Tumor size (≥ 5 cm/< 5 cm)	2	1092	Fixed	0.51(0.41-0.64)	0.000	0	0.430
Tumor number (multiple/single)	2	1170	Fixed	1.39(1.06-1.82)	0.020	0	0.650
AFP value(>400/≤400 ng/dL)	3	1494	Random	0.56(0.46-0.68)	0.006	71	0.004

CI, confidence interval; HBV, hepatitis B virus; N, number; OR, odds ratio.

PAB and BCLC stage: Three studies reported BCLC tumor stage (A–B/C), and the pooled results indicated that the proportion of patients with early BCLC stage in the high serum prealbumin level group was higher (OR = 2.23, 95% CI = 1.60–3.11, P < 0.0001) (**Table 2**).

PAB and Child-Pugh classification: Two studies reported the Child-Pugh liver function classification (A/B), and the summary results showed that in HCC patients with high serum prealbumin levels, the proportion of those with good Child-Pugh liver function was higher (OR = 5.36, 95% CI = 3.29–8.72, P < 0.0001) (**Table 2**).

PAB and vascular invasion: Two studies reported on the presence or absence of vascular invasion, and the pooled results indicated that the incidence of vascular invasion was lower in the HCC group with high serum prealbumin levels (OR = 0.44, 95% CI = 0.34–0.58, P < 0.0001) (**Table 2**).

PAB and distant metastasis: Three studies reported on distant metastasis, and the pooled results indicated that patients with high serum prealbumin levels had a lower incidence of distant metastasis in the HCC group (OR = 0.22, 95% CI = 0.14–0.35, P < 0.0001) (**Table 2**).

PAB and tumor size: Two studies provided data on tumor size (≥ 5 cm/< 5 cm). Our results indicated that the proportion of patients with smaller tumor diameters in the high serum prealbumin level group was higher (OR = 0.51, 95% CI = 0.41-0.64, P < 0.0001) (**Table 2**).

PAB and tumor count: Two studies provided data on tumor count (multiple/individual tumors). Our results indicated that the proportion of patients with a single tumor in the high serum prealbumin level group was higher (OR = 1.39, 95% CI = 1.06–1.82, P = 0.020) (**Table 2**).

PAB and AFP levels: Three studies reported AFP levels (> 400/≤ 400 ng/dL), where AFP levels ≤ 400 ng/dL were considered low. The pooled results indicated that the proportion of patients with low AFP levels was higher in the high serum prealbumin level group (OR = 0.56, 95% CI = 0.46–0.68, P = 0.006) (**Table 2**).

### 3.3 Subgroup analysis

Subgroup analyses were conducted based on the HR extraction method (directly reported vs. estimated from survival curves), sample size, and follow-up period to explore potential sources of heterogeneity (**Table 3**). Importantly, the subgroup analysis based on the HR extraction method revealed consistent results: the pooled HR was 0.57 (95% CI: 0.48–0.67, p<0.0001) for studies with HRs estimated from curves and 0.68 (95% CI: 0.61–0.76, p<0.0001) for studies with directly reported HRs. The strong agreement in the direction, significance, and overlapping confidence intervals between these subgroups indicates that the method of HR acquisition did not materially alter the primary conclusion, thereby strengthening the robustness of our findings. Furthermore, subgroup analysis based on sample size revealed that the pooled HR of studies with sample sizes ≥ 400 was 0.66 (95% CI: 0.59–0.73, p<0.0001). In studies with sample sizes less than 400 cases, the combined HR was 0.60 (95% CI: 0.49–0.73, p<0.0001). Similarly, when these studies were analyzed based on the follow-up period, the results showed that in studies with a longer follow-up period (≥ 80 months), patients with low serum prealbumin levels had significantly worse prognosis (HR = 0.65, 95% CI: 0.58–0.73, p<0.0001). This association was also observed in short-term follow-up studies (< 80 months) (HR = 0.63, 95% CI: 0.54–0.73, p<0.0001). The interaction tests for subgroup differences were not statistically significant (all P values were greater than 0.05). This indicates that the method of obtaining HR, sample size, and follow-up duration did not significantly influence the combined effect size.

**Table 3:** Subgroup analysis results of the impact of serum prealbumin on overall survival of HCC.

Parameter	N of studies	Effect model	HR(95%CI)	P-value	Heterogeneity test		The P-value for the interaction test of the differences among the subgroups
					I^2^ (%)	P-value	
Overall	8	Fixed	0.64(0.59–0.70)	0.000	0	0.53	
HR extraction method							P=0.09
report	4	Fixed	0.68(0.61–0.76)	0.000	0	0.98	
survival curve	4	Fixed	0.57(0.48–0.67)	0.000	0	0.39	
Sample size							P=0.42
≥400	4	Fixed	0.66(0.59–0.73)	0.000	0	0.53	
<400	4	Fixed	0.60(0.49–0.73)	0.000	8	0.35	
Duration of follow-up(months)							P=0.76
≥80	4	Fixed	0.65(0.58–0.73)	0.000	0	0.55	
<80	4	Fixed	0.63(0.54–0.73)	0.000	23	0.27	

### 3.4 Sensitivity analysis

A sensitivity analysis was performed using STATA version 16.0 to assess the robustness of the association between baseline serum prealbumin and OS. The one-study-removed approach confirmed that no single study disproportionately influenced the overall pooled estimate (**Supplementary File 2**). Furthermore, as detailed in the subgroup analysis above, we specifically evaluated the influence of the ‘HR extraction method’ as a key variable. The consistent association observed across both methodological subgroups further demonstrates that the pooled result is not sensitive to this particular source of potential measurement variation.

### 3.5 Publication bias

**Figure 3:**
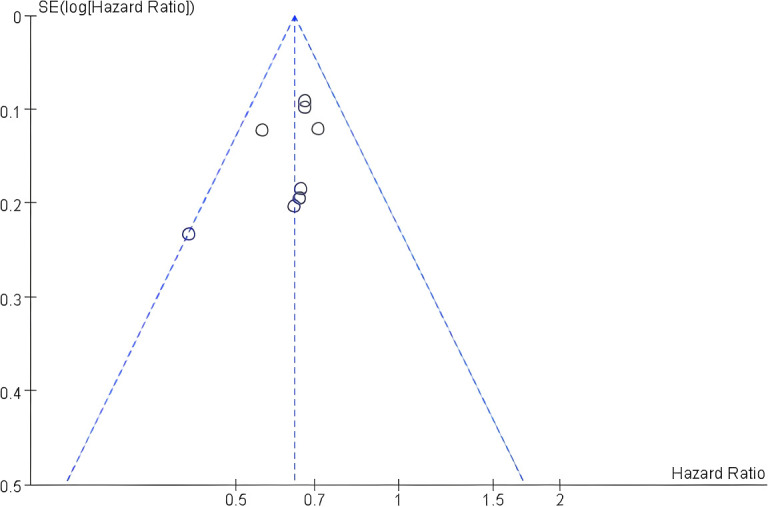
Funnel plot for assessing publication bias in OS.

The purpose of the publication bias analysis is to assess the reliability of the meta-analysis results, with particular attention to those that are statistically significant, as these are more susceptible to bias (20). Using the overall survival (OS) data, publication bias was assessed by means of a funnel plot and Egger’s regression test. Visual inspection of the funnel plot (**Figure 3**) indicated an approximately symmetrical shape, suggesting no obvious evidence of publication bias. Statistical confirmation was provided by Egger’s regression test, which yielded a non-significant result (p = 0.204). Collectively, these findings suggest that publication bias was unlikely to have substantially influenced the pooled effect estimates in this meta-analysis.

### 3.6 Evaluation of evidence quality (GRADE)

We used the GRADE (Grading of Recommendations Assessment, Development and Evaluation) method to assess the quality of evidence for the main outcome of this study: the association between low serum prealbumin levels and poorer OS in HCC patients treated with TACE. The overall quality of the evidence was rated as low (**Supplementary File 3**). The reasons for the downgrade included: 1) limitations in study design, all included studies were of retrospective observational design, but although the NOS score was relatively high, there was still a risk of uncontrolled confounding bias (downgraded by 1 level); 2) indirectness: all studies were conducted in Chinese populations, which limited the generalizability of their results to different etiologies and populations (downgraded by 1 level). Although the number of included studies was limited, the total sample size was large, and the funnel plot and Egger’s test did not indicate significant publication bias, so the downgrade due to imprecision and publication bias was not applied. The low evidence level indicates that our confidence in the estimated value of this association is limited, and more high-quality prospective studies are needed to verify it in the future.

## 4 Discussion

This study conducted a meta-analysis of eight different cohorts from five studies, involving a total of 2,996 patients with liver cancer. While some primary studies have suggested a potential link between prealbumin levels and outcomes in HCC patients receiving TACE (11–13), the evidence remains scattered and lacks quantitative synthesis. This meta-analysis, therefore, aims to consolidate these findings by providing a higher level of evidence. This research systematically investigates, for the first time, whether baseline serum prealbumin (PAB) levels can predict outcomes in HCC patients receiving TACE. The main analysis results showed that lower baseline PAB levels were significantly associated with poorer overall survival (OS) (combined HR = 0.64, 95% CI: 0.59–0.70, p < 0.0001). This association remained consistent across different subgroup analyses. Additionally, PAB levels were significantly correlated with various clinicopathological features of tumors, further supporting its potential value as a comprehensive prognostic indicator.

Serum prealbumin (PAB) is an acute-phase protein synthesized in the liver. Compared to traditional liver function indicators such as albumin, PAB has a shorter half-life (2 days versus 20 days) and can more rapidly reflect dynamic changes in the body’s nutritional status and liver synthetic function (21). The shorter half-life of PAB makes it more sensitive in assessing the overall condition of tumor patients compared to traditional indicators such as albumin (22). In recent years, several studies have identified PAB as an independent predictor of prognosis for various digestive tract malignancies, including esophageal cancer, gastric cancer, and colorectal cancer (23–28). Previous meta-analyses (10, 22) indicated that low serum PAB levels in HCC patients before surgery were significantly associated with poor postoperative prognosis. Our research results further verified this conclusion in HCC patients receiving TACE treatment, providing robust evidence for its clinical application.

The mechanisms underlying the association between PAB and the prognosis of HCC patients may involve multiple interrelated pathological and physiological processes. Firstly, PAB is a sensitive indicator reflecting the nutritional status of the body. A decrease in its level directly suggests a state of protein-energy malnutrition, which is closely related to the occurrence of cancer cachexia—a syndrome that is an important factor leading to weight loss, impaired immune function, and poor treatment outcomes (29). Experimental studies have shown that malnutrition can cause an imbalance in the ratio of CD4+/CD8+ T cells and a decrease in NK cell activity, thereby affecting the immune surveillance function of tumors (30). Secondly, as a negative acute-phase protein, the serum level of PAB is significantly suppressed under systemic inflammatory conditions. Molecular biology studies have shown that inflammatory cytokines such as IL-6 can reduce serum PAB levels by inhibiting PAB gene transcription and promoting its catabolism (31). Clinical research has found that PAB is significantly negatively correlated with C-reactive protein (CRP), and low PAB levels often indicate a persistent systemic inflammatory response (32). This chronic inflammatory microenvironment can activate signaling pathways such as NF-κB to promote tumor proliferation, invasion, and immune escape (33). Additionally, as a protein synthesized by hepatocytes, the level of PAB can also indirectly reflect liver reserve function. Patients with poor liver function have a decreased capacity to synthesize PAB (34), which may affect their tolerance to transarterial chemoembolization (TACE) treatment and postoperative recovery. Recent studies have also found that PAB may influence cell differentiation and metabolic processes by participating in the transport of vitamin A and thyroid hormones, thereby indirectly affecting these cellular functions (35). Therefore, PAB comprehensively reflects the patient’s body condition from three dimensions: nutritional status, inflammatory response, and liver function, which may be the important biological basis for its ability to predict prognosis.

Our research mainly focused on the association between serum prealbumin (PAB) and overall survival (OS), and provided robust evidence based on multiple cohort studies and meta-analyses. Moreover, when analyzing the correlation between serum prealbumin and OS, no significant heterogeneity was found (I^2^ = 0%, P = 0.53), which further supports the consistency of our findings. We first conducted stratified analysis based on the HR extraction method (including survival curve estimation or direct extraction), and performed subgroup analysis according to sample size (≥400 or <400) and follow-up time (≥80 months or <80 months). Nevertheless, all subgroup analysis results were consistent in the direction of effect, and no statistically significant heterogeneity was observed either within or between groups. This further supports the reliability and robustness of the main conclusion.

The present study was unable to conduct a meta-analysis on the relationship between PAB and progression-free survival (PFS). This is because among the five studies included in the final analysis, only one (12) reported the correlation data between PAB and PFS. Quantitative synthesis (meta-analysis) requires at least two independent studies to provide comparable effect sizes suitable for combination. The data from a single study does not meet this basic statistical requirement, so this review can only provide a qualitative description and discussion of its findings. This situation highlights a significant gap in current research in this field. The scarcity of this evidence may be due to several factors. Firstly, overall survival (OS) is regarded as the ‘gold standard’ endpoint for evaluating treatment efficacy and long-term outcomes in solid tumor prognosis studies, because it is objective, unbiased, and directly reflects the ultimate benefits of treatment, and it is often prioritized as the primary endpoint in study designs. In contrast, the assessment of PFS may be affected by follow-up frequency, differences in imaging assessment criteria, and subsequent treatments, making it more challenging to standardize extraction from retrospective data. At the same time, the prognostic mechanism of PAB, as explained in this study, may have a more direct and profound impact on patients’ long-term survival. Malnutrition and chronic inflammation can weaken patients’ physical condition, immune function, and tolerance to treatment. Although these factors also affect tumor progression, more critically, they significantly increase the risk of patients dying from non-tumor progression complications (such as infection and organ failure) (36). Therefore, as a host factor marker, the predictive efficacy of PAB for OS may precede or be stronger than that for PFS. Although only a single study supports this, its positive results provide preliminary clues for PAB predicting PFS, suggesting that low PAB levels may also be associated with earlier disease progression. This finding is consistent with the results observed in other tumor types (25). To conduct a more comprehensive evaluation of the prognostic value of PAB, we strongly recommend that future prospective studies incorporate PFS and objective response rate (ORR) as key endpoint indicators in their design. This approach will accumulate sufficient data and enable future assessment of the role of PAB in predicting tumor progression and treatment response.

In addition, we also analyzed the correlation between the PAB level and the clinicopathological features of the tumor, including HBV infection, serum AFP level, Barcelona Clinic Liver Cancer (BCLC) stage, vascular invasion, distant metastasis, number of tumors, and tumor size, which are strongly associated with the invasiveness and metastasis of HCC (37). The results showed that a low serum prealbumin level was closely related to the clinicopathological features representing the invasion and metastasis of HCC, including a later BCLC stage, worse Child-Pugh liver function classification, vascular invasion, distant metastasis, multiple tumors, larger tumor size, and higher AFP level. This is consistent with the research results of Lei (13), Hou (12), and et al.

In recent years, various inflammation-related biomarkers (such as NLR, PLR, and SII) have been confirmed to be associated with the prognosis of HCC (38, 39). However, these biomarkers require the calculation of multiple blood parameters. There is considerable controversy over their cutoff values. PAB, as a single indicator, is both simple and reliable, and may have greater clinical significance in practice. It is worth noting that recent studies have begun to explore the combination of PAB and other inflammatory biomarkers. For example, the ratios of PAB to CRP and PAB to fibrinogen have better predictive value than single indicators (23, 26). Additionally, the combined measurement of PAB with serum total bile acid (TBA) and cholinesterase (CHE) also shows good application prospects (40). Nevertheless, most of the current related studies are still at the stage of retrospective analysis. Large-scale prospective studies are needed to verify the clinical applicability of these composite indicators.

In addition, future research should explore the integrative value of PAB with existing liver function reserve assessment systems (such as ALBI score) or tumor efficacy evaluation standards (such as mRECIST). For instance, the ALBI score is recognized as an effective tool for evaluating liver function and prognosis in patients with hepatocellular carcinoma (41), and PAB, as a direct reflection of nutritional and synthetic functions, may have a complementary relationship with it. Combining PAB analysis with composite indicators reflecting the systemic inflammatory state (such as SII) may help construct a more comprehensive host status assessment model (42). Regarding efficacy evaluation, exploring the correlation between the dynamic changes of PAB before and after treatment and the imaging response based on the mRECIST standard may provide a blood biomarker basis for judging the biological effect of TACE treatment in addition to imaging (43). Although PAB as a single indicator has the prominent advantage of simplicity and ease of use, its collaborative application with multi-dimensional scoring systems may be an important direction for achieving more precise individualized prognostic assessment.

The results of this study have significant clinical significance. Firstly, we suggest including PAB in the routine assessment system for HCC patients before TACE treatment, in order to more accurately identify high-risk patients. Specifically, future research could focus on establishing the threshold values of PAB for clinical decision-making. For instance, future studies could explore a specific PAB threshold (such as ≤ 170 mg/L or below the lower limit of the normal range) to identify patient groups with extremely high risk of post-TACE complications due to severe malnutrition and insufficient liver function reserve, or those who may not benefit from TACE. Combining this threshold with liver function Child-Pugh classification, ALBI score, and other relevant indicators may help optimize the patient selection strategy for TACE treatment. Regarding nutritional intervention, low PAB levels (e.g., below the aforementioned threshold) can serve as a clear indication for initiating intensive nutritional support. The European Society for Clinical Nutrition and Metabolism (ESPEN) guidelines recommend early nutritional intervention for cancer patients with malnutrition (44). For HCC patients with low PAB, the clinical management pathway could be designed as follows: once identified, intervention by a nutritionist is initiated immediately and an individualized nutritional plan is formulated. Changes in PAB levels during the TACE perioperative period and subsequent treatments are continuously monitored to assess the intervention effect and dynamically adjust the plan. Using PAB as a ‘trigger point’ and ‘monitoring scale’ for nutritional intervention is expected to achieve a closed loop from risk identification to active management. Secondly, exploring whether anti-inflammatory therapy can improve prognosis for patients with low PAB is also a direction worthy of in-depth research. Recent basic studies have shown that targeting the IL-6/JAK/STAT3 signaling pathway may reverse cancer-related inflammatory states (45). Clinical studies have also found that anti-inflammatory drugs such as COX-2 inhibitors may improve the prognosis of cancer patients (46). In terms of treatment strategies, low PAB patients may need more individualized multidisciplinary comprehensive management. For these high-risk patients, targeted therapy, immunotherapy, or other systemic treatments can be considered in combination with TACE. For example, the recently published IMbrave150 study (22) showed that atezolizumab combined with bevacizumab can significantly improve the survival of patients with unresectable HCC. Future research can explore whether low PAB patients can benefit more from this combined treatment compared to patients with normal PAB levels.

This study has several methodological advantages. Firstly, we systematically searched multiple databases in both Chinese and English, minimizing publication bias to the greatest extent. Secondly, we included a large sample size (totaling nearly 3,000 patients), enhancing the statistical power. Thirdly, we adopted strict literature quality evaluation standards to ensure the scientific rigor of the included studies. Fourthly, we conducted detailed subgroup and sensitivity analyses, demonstrating good stability of the results. Fifthly, this study was the first to systematically evaluate the relationship between prealbumin (PAB) and various clinicopathological characteristics of multiple tumors, providing more comprehensive evidence.

However, this study also has several limitations. Firstly, all included studies were retrospective in design and, therefore, carry inherent risks of selection bias. Secondly, all included studies were conducted in China, which limits the generalizability of our findings to populations with different etiologies (e.g., alcohol- or NASH-related HCC), genetic backgrounds, and healthcare systems. Although we comprehensively searched both Chinese and English databases to minimize language bias, we cannot rule out the possibility of missing relevant studies published in other languages. Thirdly, the limited number of included studies constrained the power of our subgroup analyses and precluded a meaningful meta-regression to explore the potential moderating effect of varying PAB cutoff values (ranging from 159 to 190 mg/L), which is an important factor for clinical application and warrants standardization in future studies. Fourthly, the HRs and CIs for some included studies were estimated from published survival curves. Although this is an accepted method for secondary data synthesis (19), it can introduce measurement error due to graph digitization and model assumptions. While our subgroup analysis showed highly consistent results regardless of the HR extraction method, which mitigates concerns about this bias substantially affecting the main conclusion, interpretation of the individual effect sizes from these specific studies should be made with caution. Fifthly, despite our extensive retrieval strategies and manual screening, we cannot completely rule out unpublished negative results. Additionally, studies that lack prealbumin as a core keyword may be missed by keyword-based searches. This inherent limitation of database retrieval may lead to selection bias. However, our comprehensive retrieval approach is designed to mitigate this risk. Sixthly, since only one study reported the relationship between PAB and progression-free survival (PFS), no definite conclusion can be drawn regarding the predictive efficacy of PAB for disease progression. Seventhly, although the overall quality of the included studies was high (with NOS scores of 7–8), none achieved full marks for cohort comparability. This reflects the inherent limitations of retrospective observational studies regarding control of confounding factors. Furthermore, some studies had deficiencies in reporting “sufficient follow-up.” Although this might have limited impact on analyses based on hard endpoints (death), it indicates a potential risk of loss-to-follow-up bias.

In conclusion, this meta-analysis based on the Chinese population indicates that the baseline serum prealbumin level is a important potential prognostic biomarker for patients with hepatocellular carcinoma undergoing TACE treatment. Its low level is significantly associated with poorer overall survival and more aggressive tumor characteristics. The PAB test is simple, rapid, and cost-effective. Considering its inclusion in routine assessment systems may help identify high-risk patients and guide clinical decisions. However, given the limitations of this study, more large-scale, prospective studies from diverse regions are needed to further verify this association and clarify its application value in clinical practice.

## Data Availability

The original contributions presented in the study are included in the article/**Supplementary Material**. Further inquiries can be directed to the corresponding author.
