# Supplementary material for: The clinical utility of serum prealbumin levels as a prognostic marker in patients with hepatocellular carcinoma undergoing transcatheter arterial chemoembolization: a meta-analysis of 2,996 patients
Source: Front Oncol. 2026 Mar 12;16:1782104. doi: 10.3389/fonc.2026.1782104 (PMC13019482; doi:10.3389/fonc.2026.1782104)
Supplement: Supplementary file 1 [file DataSheet1.pdf]

Supplementary Material List

Supplementary file 1: The search strategy.

Databases	Searching strategy	Total
PubMed	("Liver Neoplasms"[MeSH Terms] OR "carcinoma, hepatocellular"[MeSH Terms] OR "Hepatocellular Carcinoma"[Title/Abstract] OR "HCC"[Title/Abstract] OR "Liver Cancer"[Title/Abstract] OR "hepatoma"[Title/Abstract]) AND ("chemoembolization, therapeutic"[MeSH Terms] OR "Transarterial Chemoembolization"[Title/Abstract] OR "TACE"[Title/Abstract] OR "chemoembolization"[Title/Abstract]) AND ("Prealbumin"[MeSH Terms] OR"Prealbumin"[Title/Abstract] OR "PAB"[Title/Abstract] OR "Transthyretin"[Title/Abstract] OR "Nutrition Assessment"[MeSH Terms] OR "Nutritional Status"[MeSH Terms] OR "prognostic nutrition"[Title/Abstract] OR "nutritional index"[Title/Abstract])	43
Web of science	1: TS=(hepatocellular carcinoma OR HCC OR liver cancer OR hepatoma)<br>2: TS=(transarterial chemoembolization OR TACE OR chemoembolization)<br>3: TS=(prealbumin OR PAB OR transthyretin OR nutrition assessment OR nutritional status OR prognostic nutrition OR nutritional index)<br>4: #3 AND #2 AND #1	77
Embase	#4. AND #2 AND #3<br>#3. 'prealbumin'/exp OR 'prealbumin':ti,ab OR 'pab':ti,ab OR 'transthyretin':ti,ab OR 'nutrition assessment'/exp OR 'nutrition status'/exp OR 'prognostic nutrition':ti,ab OR 'nutritional index':ti,ab<br>#2. 'transarterial chemoembolization'/exp OR 'chemoembolization'/exp OR 'tace':ti,ab OR 'transarterial chemoembolization':ti,ab<br>#1. 'liver cell carcinoma'/exp OR 'liver tumor'/exp OR 'hepatocellular carcinoma':ti,ab OR 'hcc':ti,ab OR 'liver cancer':ti,ab	144
Cochrane	#1 MeSH descriptor: [Liver Neoplasms] explode all trees<br>#2 MeSH descriptor: [Carcinoma, Hepatocellular] explode all trees<br>#3 (hepatocellular carcinoma):ti,ab,kw OR (HCC):ti,ab,kw OR (liver cancer):ti,ab,kw (Word variations have been searched)<br>#4 #1 OR #2 OR #3<br>#5 MeSH descriptor: [Chemoembolization, Therapeutic] explode all trees<br>#6 (transarterial chemoembolization):ti,ab,kw OR (TACE):ti,ab,kw OR (chemoembolization):ti,ab,kw (Word variations have been searched)<br>#7 #5 OR #6<br>#8 MeSH descriptor: [Prealbumin] explode all trees<br>#9 MeSH descriptor: [Nutritional Status] explode all trees<br>#10 MeSH descriptor: [Nutrition Assessment] explode all trees<br>#11 (prealbumin):ti,ab,kw OR (PAB):ti,ab,kw OR (transthyretin):ti,ab,kw OR (nutritional status):ti,ab,kw OR (nutrition assessment):ti,ab,kw OR (prognostic nutrition):ti,ab,kw OR (nutritional index):ti,ab,kw(Word variations have been searched)<br>#12 #8 OR #9 OR #10 OR #11	7

	#13 #4 AND #7 AND #12	
CNKI	（主题：'肝细胞癌' + '肝癌' + 'HCC'）AND（主题：'经动脉化疗栓塞' + 'TACE' + '化疗栓塞'）AND（主题：'前白蛋白' + 'PAB' + '转甲状腺素蛋白' + '营养状态' + '营养状况' + '营养评估' + '预后营养' + '营养指数'）	92
Wanfang	（主题：'肝细胞癌' + '肝癌' + 'HCC'）AND（主题：'经动脉化疗栓塞' + 'TACE' + '化疗栓塞'）AND（主题：'前白蛋白' + 'PAB' + '转甲状腺素蛋白' + '营养状态' + '营养状况' + '营养评估' + '预后营养' + '营养指数' ）	163
VIP	((((题名或关键词=肝细胞癌 OR 题名或关键词=肝癌) OR 题名或关键词=HCC) AND ((题名或关键词=经动脉化疗栓塞 OR 题名或关键词=TACE) OR 题名或关键词=化疗栓塞)) AND (((((((题名或关键词=营养状态 OR 题名或关键词=营养状况) OR 题名或关键词=营养评估) OR 题名或关键词=预后营养) OR 题名或关键词=营养指数) OR 题名或关键词=前白蛋白) OR 题名或关键词=PAB) OR 题名或关键词=转甲状腺素蛋白))	34
CBM	("经动脉化疗栓塞"[常用字段:智能] OR "TACE"[常用字段:智能] OR "化疗栓塞"[常用字段:智能]) AND ("肝细胞癌"[常用字段:智能] OR "肝癌"[常用字段:智能] OR "HCC"[常用字段:智能]) AND ("前白蛋白"[常用字段:智能] OR "Prealbumin"[常用字段:智能] OR "PAB"[常用字段:智能] OR "Transthyretin"[常用字段:智能] OR "转甲状腺素蛋白"[常用字段:智能] OR "营养状态"[常用字段:智能] OR "营养状况"[常用字段:智能] OR "营养评估"[常用字段:智能] OR "营养指数"[常用字段:智能] OR "预后营养"[常用字段:智能])	103

Supplementary file 2: Leave-one-out sensitivity analysis for the association between serum prealbumin and overall survival.

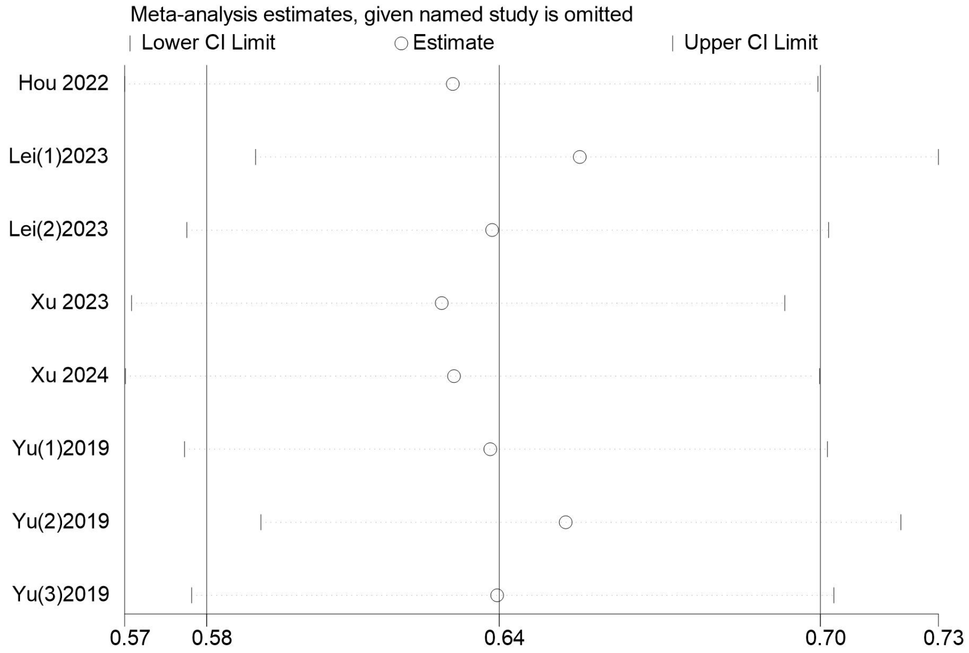

Supplementary file 3: GRADE Assessment.

Table S1. Summary of Findings (SoF) for serum prealbumin and overall survival (OS) in HCC patients undergoing TACE

Population: Patients with hepatocellular carcinoma (HCC) undergoing transcatheter arterial chemoembolization (TACE)
Comparison: High serum prealbumin vs. Low serum prealbumin
Outcome: Overall survival (OS)

**Outcome**	**No. of studies**	**Total participants**	**Pooled effect (95% CI)**	**I^2**	**Quality (GRADE)**	**Reasons for downgrade/upgrade**
Overall Survival (OS)	5 retrospective observational studies	2996	HR=0.64 (0.59–0.70)	0%	⊕⊕◯◯Low	Initial level: Low; 1 downgrade for selection bias and possible publication bias; Upgraded 1 level for large sample, consistent effect, and no heterogeneity

Table S2. GRADE Evidence Profile for serum prealbumin and overall survival (OS)

Study design: 5 retrospective observational studies

Total patients: 2996

Pooled HR: 0.64 (95% CI 0.59–0.70)

Domain	Assessment	Justification
**Study design**	Observational (retrospective cohort)	All 5 studies were retrospective observational studies
**Initial evidence level**	Low	Observational evidence starts at Low quality
**1. Risk of bias**	Medium Risk of Bias(-1 level)	Given that all the studies have inherent risks of selection bias due to their retrospective design, and all the studies are from China, although the possibility of publication bias has been evaluated and found not to be significant, it cannot be completely ruled out.
**2. nconsistency**	No serious limitation	I^2 = 0%, highly consistent results
**3. Indirectness**	No serious limitation	Direct population, exposure, and outcome
**4. Imprecision**	No serious limitation	Large sample (n=2996) with narrow 95% CI
**5.Publication bias**	No serious limitation	No asymmetry; no small-study effect
**Upgrade factors**	+1 level	Large sample, strong consistent effect, no heterogeneity
**Final evidence quality**	⊕ ⊕◯ ◯Low	Low + 1 downgrade + 1 upgrade = Low

Heterogeneity: I^2 = 0%
